# Supplementary material for: Shape Evolution in Two Acts: Morphological Diversity of Larval and Adult Neoaustraranan Frogs
Source: Animals (Basel). 2024 May 8;14(10):1406. doi: 10.3390/ani14101406 (PMC11117230; doi:10.3390/ani14101406)
Supplement: Supplementary file 1 [file animals-14-01406-s001.zip › SuppFigS1.pdf]

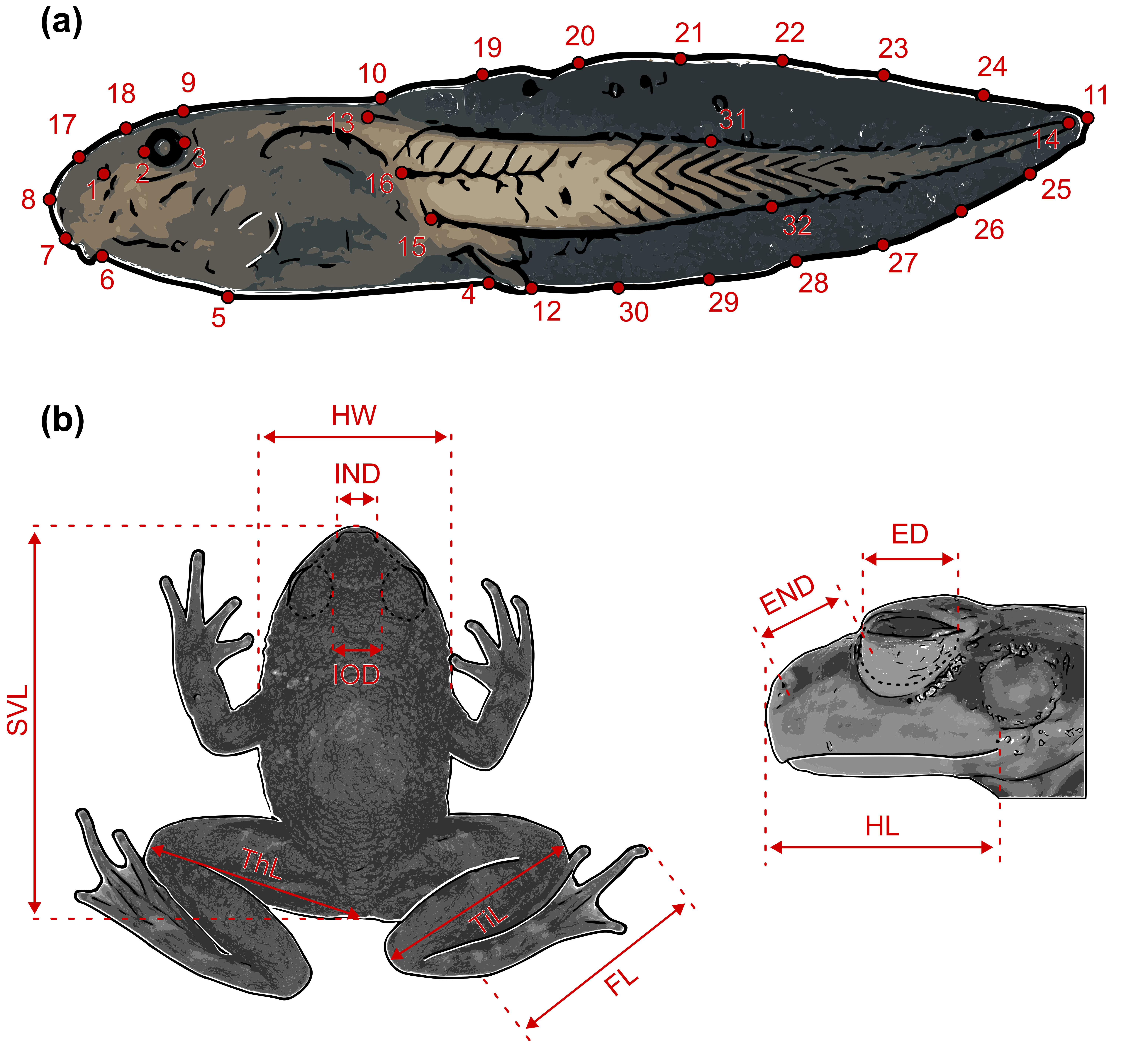

Figure S1. Landmark configuration and linear measures defined to represent shape in tadpoles and adults of Neoaustrarana. **(a)** Landmarks: 1 nostril, 2 anterior edge of eye, 3 posterior edge of eye, 4 base of vent tube, 5 maximum ventral curvature of body, 6 posterior edge of oral disc, 7 anterior edge of oral disc, 8 tip of snout, 9 maximum dorsal curvature of body, 10 origin of dorsal fin, 11 tip of tail, 12 origin of ventral fin, 13 origin of dorsal caudal musculature, 14 tip of caudal musculatur e, 15 origin of ventral musculature, 16 anterior point of caudal myotome intersection, 17–18 equidistant points between landmarks 8 and 9, 19–24 equidistant points between landmarks 10 and 11, 25 –30 equidistant points between landmarks 11 and 12, 31–32 equidistant points between landmarks 13 and 14 and 14 and 15 respectively;**(b)** Linear measurements: ED eye diameter, END eye–nostril distance, HL head length, HW head width, IND internarial distance, IOD interorbital distance, FL foot length, SVL snout–vent length, ThL thigh length, TiL tibia length.
