# Supplementary material for: Shape Evolution in Two Acts: Morphological Diversity of Larval and Adult Neoaustraranan Frogs
Source: Animals (Basel). 2024 May 8;14(10):1406. doi: 10.3390/ani14101406 (PMC11117230; doi:10.3390/ani14101406)
Supplement: Supplementary file 1 [file animals-14-01406-s001.zip › SuppFigS2.pdf]

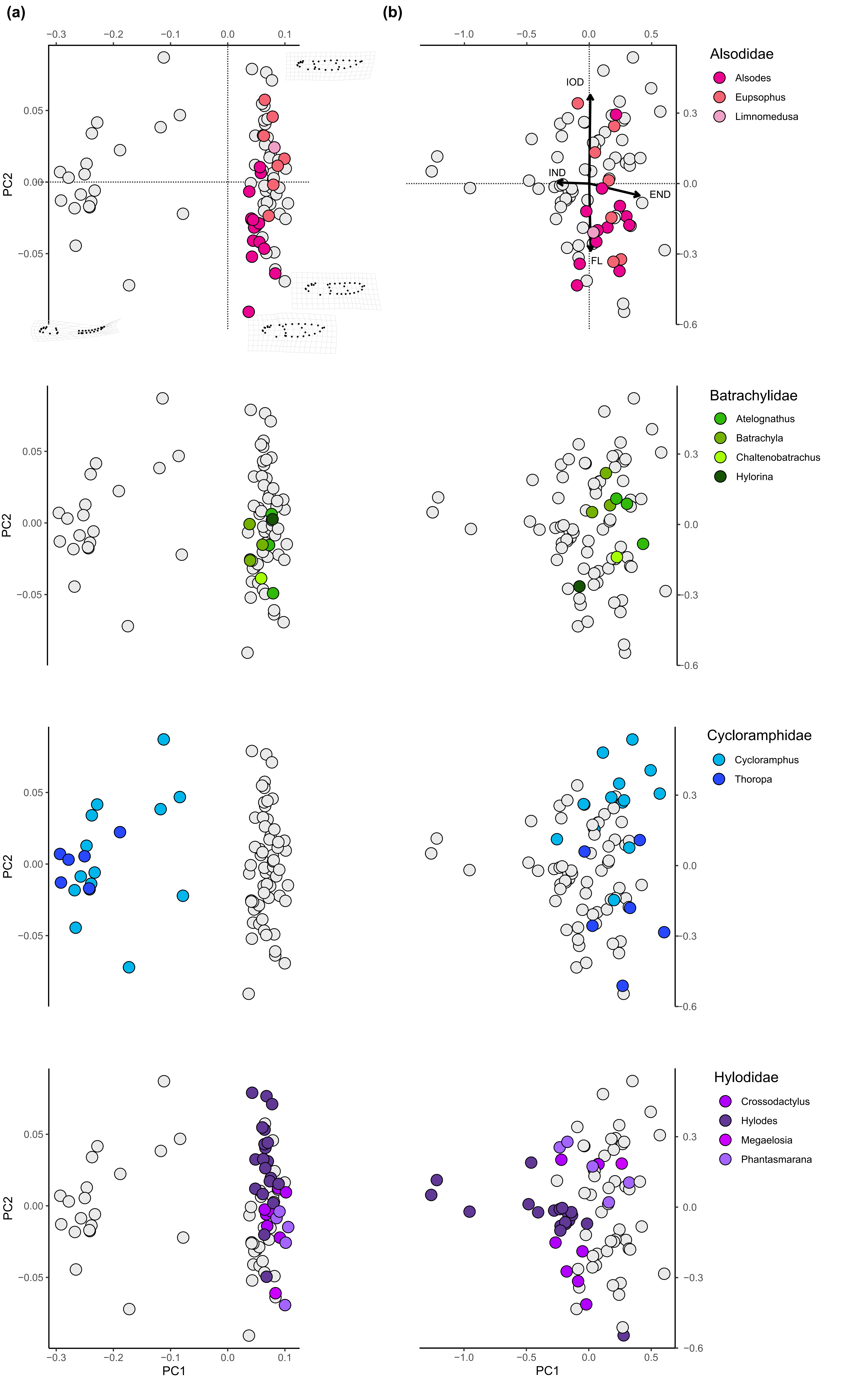

Figure S2. Larval **(a)** and adult **(b)** morphospaces for Neoaustrarana, discriminated by family to highlight genus distribution. Results of principal component analyses are shown, with species ordination on the two first principal components (PC). Deformation grids on larval morphospace illustrate shape change along axes, whereas arrows in the adult biplot are linear variables with higher correlations to axes. END eye-nostril-distance, IND internarial distance, IOD interorbital distance, FL foot length.
