# Supplementary material for: Shape Evolution in Two Acts: Morphological Diversity of Larval and Adult Neoaustraranan Frogs
Source: Animals (Basel). 2024 May 8;14(10):1406. doi: 10.3390/ani14101406 (PMC11117230; doi:10.3390/ani14101406)
Supplement: Supplementary file 1 [file animals-14-01406-s001.zip › SuppFigS3.pdf]

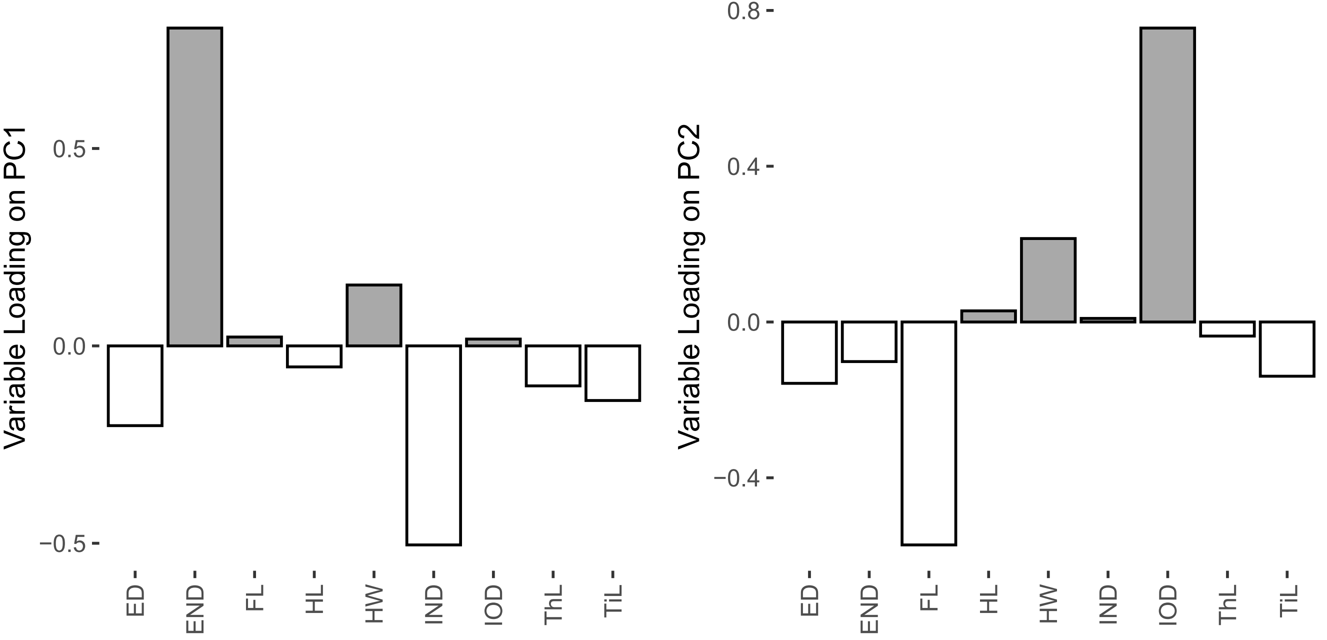

Figure S3. Linear measurements registered in adult frogs and their correlations with principal components 1 and 2 of PC analysis. Linear measurements: ED eye diameter, END eye–nostril distance, HL head length, HW head width, IND internarial distance, IOD interorbital distance, FL foot length, SVL snout–vent length, ThL thigh length, TiL tibia length.
