# Supplementary material for: Shape Evolution in Two Acts: Morphological Diversity of Larval and Adult Neoaustraranan Frogs
Source: Animals (Basel). 2024 May 8;14(10):1406. doi: 10.3390/ani14101406 (PMC11117230; doi:10.3390/ani14101406)
Supplement: Supplementary file 1 [file animals-14-01406-s001.zip › SuppFigS4.pdf]

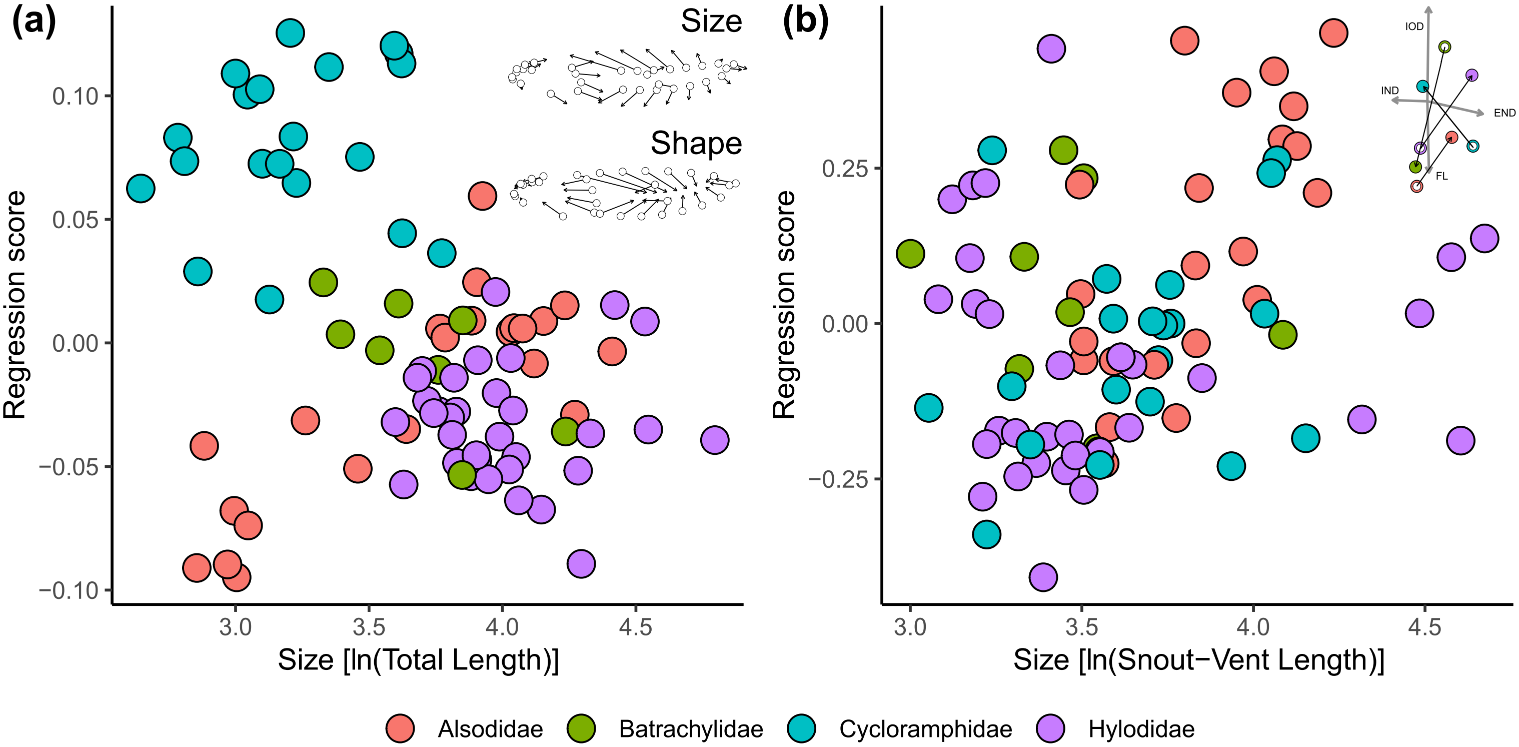

Figure S4. Allometric patterns in tadpoles **(a)** and adults **(b)** of Neoaustrarana. Shape is represented with raw distribution of regression scores (the projection of data points onto an axis in the direction of the regression vector). Deformation schemes depict shape changes a long the axes, tending to higher body/tails in large larvae, and to flattened shapes with increasin g PC1 score. In adults, allometric patterns can be interpreted from the biplot at the upper right, where the smallest (empty circles) and largest (solid circles) species are shown, and morphological changes are hinted by their ordination regarding relevant linear measurements. END eye-nostril distance, IND internarial distance, IOD interorbital distance, FL foot length .
