# Supplementary material for: Shape Evolution in Two Acts: Morphological Diversity of Larval and Adult Neoaustraranan Frogs
Source: Animals (Basel). 2024 May 8;14(10):1406. doi: 10.3390/ani14101406 (PMC11117230; doi:10.3390/ani14101406)
Supplement: Supplementary file 1 [file animals-14-01406-s001.zip › SuppFigS5.pdf]

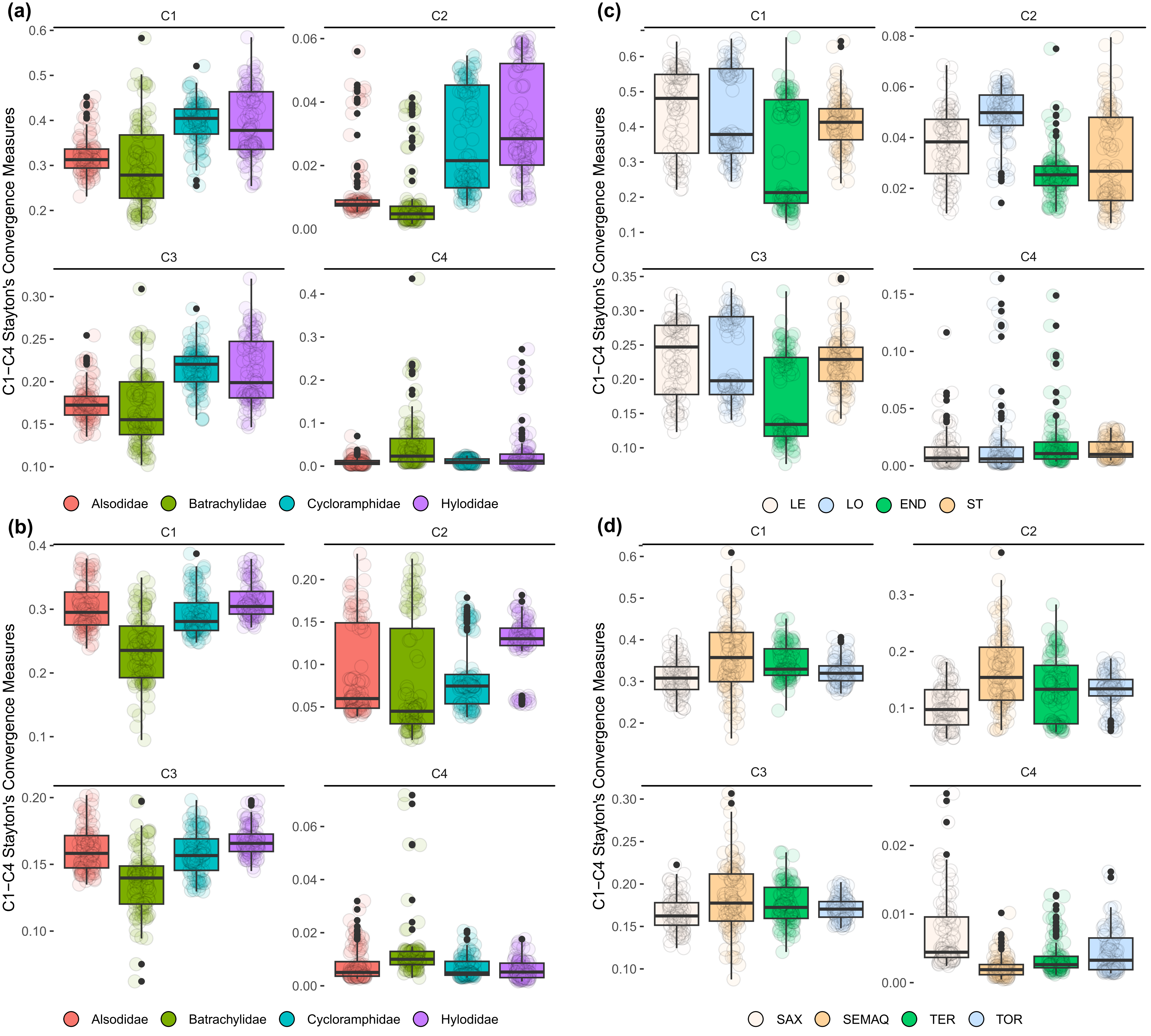

Figure S5. Uncertainty of shape convergence in neoaustraranan tadpoles **(a, c)** and adults **(b, d)**, assessed by Stayton's C1-C4 values. LE lentic, LO lotic, END endotrophic, ST semiterrestrial, SAX saxicolous, SEMAQ semi-aquatic, TER terrestrial, TOR torrential.
