# Supplementary material for: Shape Evolution in Two Acts: Morphological Diversity of Larval and Adult Neoaustraranan Frogs
Source: Animals (Basel). 2024 May 8;14(10):1406. doi: 10.3390/ani14101406 (PMC11117230; doi:10.3390/ani14101406)
Supplement: Supplementary file 1 [file animals-14-01406-s001.zip › TableS1.pdf]

Table S1. List of species and data sources used for the present study. Species were classified taxonomically (Genus, Family) and according to larval functional groups (Guild) and adults (Ecomorph). For geometric morphometrics, we indicate which tadpoles were photographed (Voucher) and images obtained from the literature; for obtaining linear measurements, we indicate animals photographed for the study (Voucher), species whose measurements were taken in ImageJ from figures of previous studies, and measurements obtained from the text/tables in these studies. References are provided afterward. LE lentic, LO lotic, END endotrophic, ST semiterrestrial, SAX saxicolous, SEMAQ semi-aquatic, TER terrestrial, TOR torrential.

Species	Genus	Family	Tadpoles			Adults				References
			Source: GM[1]	Voucher	Guild	Source LM[2]	Voucher	Sex	Ecomorph	
Alsodes barrioi	*Alsodes*	Alsodidae	Photo	FML 04698.1; FML 04594	LO	ImageJ, figure	-	?	TER	[1–7]
Alsodes coppingeri	*Alsodes*	Alsodidae	Literature	-	LO	ImageJ	LGE25458	M	TER	[5,6,8]
Alsodes gargola	*Alsodes*	Alsodidae	Literature	-	LE	ImageJ, figure	-	M	SEMAQ	[3,5,9,10]
Alsodes hugoi	*Alsodes*	Alsodidae	Literature	-	LO	ImageJ, figure	-	M	TOR	[5,11,12]
Alsodes igneus	*Alsodes*	Alsodidae	Literature	-	LO	ImageJ, figure	-	M	TER	[5,6,13]
Alsodes montanus	*Alsodes*	Alsodidae	Literature	-	LO	ImageJ, figure	-	M	TOR	[2,5,12,14–16]
Alsodes neuquensis	*Alsodes*	Alsodidae	Literature	-	LE	Literature	-	M	SEMAQ	[5,17]
Alsodes nodosus	*Alsodes*	Alsodidae	Literature	-	LO	Literature	-	M	TOR	[2,5,12,18,19]
Alsodes norae	*Alsodes*	Alsodidae	Literature	-	LO	ImageJ, figure	-	M	TER	[6,20,21]
Alsodes pehuenche	*Alsodes*	Alsodidae	Literature	-	LE	Literature	-	M	SEMAQ	[5,17,22–24]
Alsodes tumultuosus	*Alsodes*	Alsodidae	Literature	-	LO	ImageJ, figure	-	F	SEMAQ	[2,5,12,25]
Alsodes valdiviensis	*Alsodes*	Alsodidae	Photo, literature	LGE 25515	LO	ImageJ	LGE 25507	M	TER	[2,5,6,14,26]
Alsodes vanzolinii	*Alsodes*	Alsodidae	Literature	-	LE	ImageJ, figure	-	M	TER	[5,12,27,28]
Alsodes verrucosus	*Alsodes*	Alsodidae	Literature	-	LO	ImageJ, figure	-	M	TER	[5,6,27,29]
Atelognathus nitoi	*Atelognathus*	Batrachylidae	Literature	-	LE	Literature	-	M	TER	[30–35]
Atelognathus patagonicus	*Atelognathus*	Batrachylidae	Photo, literature	LGE 21732	LE	ImageJ	FML2907-3	?	SEMAQ	[3,23,36–38]
Atelognathus reverberii	*Atelognathus*	Batrachylidae	Photo, literature	FML 04592	LE	ImageJ	FML2976	?	TER	[3,23,37,39]
Batrachyla antartandica	*Batrachyla*	Batrachylidae	Literature	-	LE	Literature	-	?	TER	[6,34,37,40,41]
Batrachyla leptopus	*Batrachyla*	Batrachylidae	Literature	-	LE	ImageJ, figure	-	M	TER	[6,34,37,40,42]
Batrachyla taeniata	*Batrachyla*	Batrachylidae	Literature	-	LE	ImageJ	FML2797	?	TER	[6,34,37,40,43]
Chaltenobatrachus grandisonae	*Chaltenobatrachus*	Batrachylidae	Literature	-	LE	Literature	-	M	SEMAQ	[6,44]
Crossodactylus caramaschii	*Crossodactylus*	Hylodidae	Photo, literature	DZSJRP 946.2	LO	ImageJ, figure	-	M	SEMAQ	[45–47]
Crossodactylus cyclospinus	*Crossodactylus*	Hylodidae	Literature	-	LO	Literature	-	M	TOR	[46,48]
Crossodactylus gaudichaudii	*Crossodactylus*	Hylodidae	Literature	-	LE	ImageJ	USNM15481	M	TOR	[3,46,49–53]
Crossodactylus schmidti	*Crossodactylus*	Hylodidae	Photo	LGE 9710; MTR II H 218|265	LO	Literature	-	M	TOR	[3,46,54–56]
Crossodactylus timbuhy	*Crossodactylus*	Hylodidae	Literature	-	LE	Literature	-	M	SEMAQ	[46,57]
Crossodactylus trachystomus	*Crossodactylus*	Hylodidae	Photo, literature	DZSJRP 2415.4	LO	Literature	-	M	TOR	[46,50,58–62]

Crossodactylus werneri	*Crossodactylus*	Hylodidae	Literature	-	LE	Literature	-	M	TOR	[46,57,63]
Cycloramphus acangatan	*Cycloramphus*	Cycloramphidae	Photo	ZUFABC sn	END	ImageJ, figure	-	M	TER	[64–66]
Cycloramphus bandeirensis	*Cycloramphus*	Cycloramphidae	Literature	-	ST	ImageJ	ZUEC 24101	M	SAX	[64,66,67]
Cycloramphus boraceiensis	*Cycloramphus*	Cycloramphidae	Photo, literature	DZSJRP 2536.4; DZSJRP 2609.4	ST	ImageJ	ZUEC 22734	M	SAX	[64,66,68–71]
Cycloramphus brasiliensis	*Cycloramphus*	Cycloramphidae	Literature	-	ST	ImageJ	CFBH22601	M	SAX	[64,66,68,70]
Cycloramphus carvalhoi	*Cycloramphus*	Cycloramphidae	Photo, literature	ZUFABC 36	END	ImageJ	UFJF692	?	TER	[64,147]
Cycloramphus dubius	*Cycloramphus*	Cycloramphidae	Photo	CHRP 2742	ST	ImageJ	ZUEC 6384	M	SAX	[64,66]
Cycloramphus eleutherodactylus	*Cycloramphus*	Cycloramphidae	Photo	ZUFABC 229	END	ImageJ	MZUSP 86823	M	TER	[64,66,68,72]
Cycloramphus fuliginosus	*Cycloramphus*	Cycloramphidae	Literature	-	ST	ImageJ	ZUEC 20908	M	SAX	[3,64,66,68,70,73]
Cycloramphus izecksohni	*Cycloramphus*	Cycloramphidae	Literature	-	ST	ImageJ	CFBH 6553	M	SAX	[64,66,70,74]
Cycloramphus lutzorum	*Cycloramphus*	Cycloramphidae	Photo, literature	DZSJRP 3050.1	ST	ImageJ	ZUEC 1719	M	SAX	[64,70,75]
Cycloramphus parvulus	*Cycloramphus*	Cycloramphidae	Photo	MUZUSP 79453	END	ImageJ	ZUEC 8073	M	TER	[64,147,148]
Cycloramphus rhyakonastes	*Cycloramphus*	Cycloramphidae	Literature	-	ST	ImageJ	ZUEC20139	M	SAX	[64,70,76]
Cycloramphus stejnegeri	*Cycloramphus*	Cycloramphidae	Photo, literature	MUZUSP 58582	END	ImageJ	MZUSP 53402	M	TER	[3,66,68,73,77,78]
Cycloramphus valae	*Cycloramphus*	Cycloramphidae	Photo, literature	MZUSP 59282	ST	ImageJ	MZUSP 59274	M	SAX	[64,66,70,79]
Eupsophus altor	*Eupsophus*	Alsodidae	Photo, literature	LGE 25516	END	ImageJ	LGE25418	?	TER	[6,80]
Eupsophus calcaratus	*Eupsophus*	Alsodidae	Photo, literature	FML 15859	END	Literature	-	M	TER	[6,81–84]
Eupsophus emiliopugini	*Eupsophus*	Alsodidae	Photo, literature	LGE 25517	END	ImageJ	LGE25506	?	TER	[6,82,85,86]
Eupsophus nahuelbutensis	*Eupsophus*	Alsodidae	Literature	-	END	ImageJ, figure	-	F	TER	[6,82,87]
Eupsophus roseus	*Eupsophus*	Alsodidae	Literature	-	END	ImageJ	FML2952-3	?	TER	[6,82,88]
Eupsophus septentrionalis	*Eupsophus*	Alsodidae	Literature	-	END	Literature	-	M	TER	[6,82,89,90]
Eupsophus vertebralis	*Eupsophus*	Alsodidae	Literature	-	END	ImageJ	FML30638	M	TER	[6,82,91]
Hylodes amnicola	*Hylodes*	Hylodidae	Literature	-	LO	Literature	-	M	SEMAQ	[92–94]
Hylodes asper	*Hylodes*	Hylodidae	Photo	ZUFABC DAS AFr8NotP1	LO	Literature	-	M	TOR	[93–97]
Hylodes babax	*Hylodes*	Hylodidae	Literature	-	LO	Literature	-	M	TOR	[60,93,94,98,99]
Hylodes caete	*Hylodes*	Hylodidae	Photo, literature	ZUFABC DAS BNa5NotP1	LO	Literature	-	M	TOR	[93,100,101]
Hylodes charadranaetes	*Hylodes*	Hylodidae	Literature	-	LO	Literature	-	M	TOR	[93,94,102]
Hylodes dactylocinus	*Hylodes*	Hylodidae	Literature	-	LO	ImageJ, figure	-	M	TOR	[93,103–105]
Hylodes fredi	*Hylodes*	Hylodidae	Literature	-	LO	Literature	-	M	SAX	[52,53,93,94,106]
Hylodes heyeri	*Hylodes*	Hylodidae	Photo, literature	DZSJRP 852.1	LO	Literature	-	M	TOR	[93,94,104,107,108]
Hylodes japi	*Hylodes*	Hylodidae	Literature	-	LO	Literature	-	M	TOR	[93,109,110]
Hylodes magalhaesi	*Hylodes*	Hylodidae	Photo, literature	DZSJRP 1483.3	LO	ImageJ, figure	-	M	TOR	[93,104,111,112]
Hylodes meridionalis	*Hylodes*	Hylodidae	Literature	-	LO	Literature	-	M	TOR	[93,94,104,113,114]
Hylodes nasus	*Hylodes*	Hylodidae	Literature	-	LO	Literature	-	M	SAX	[93,94,109,115,116]

Hylodes ornatus	*Hylodes*	Hylodidae	Literature	-	LO	Literature	-	M	TOR	[93,94,117,118]
Hylodes otavioi	*Hylodes*	Hylodidae	Literature	-	LO	Literature	-	M	TOR	[93,94,119]
Hylodes perere	*Hylodes*	Hylodidae	Literature	-	LO	Literature	-	M	TOR	[93,120]
Hylodes perplicatus	*Hylodes*	Hylodidae	Photo, literature	DZSJRP 3035.1	LO	Literature	-	M	TOR	[93,94,121]
Hylodes phyllodes	*Hylodes*	Hylodidae	Photo, literature	DZSJRP 2563.1	LO	Literature	-	M	TOR	[69,93,94,104,122,123]
Hylodes pipilans	*Hylodes*	Hylodidae	Literature	-	LO	Literature	-	M	TOR	[93,94,105,124,125]
Hylodes sazimai	*Hylodes*	Hylodidae	Photo, literature	DZSJRP 2339.2	LO	Literature	-	M	TOR	[93,94,104,126]
Hylodes uai	*Hylodes*	Hylodidae	Literature	-	LO	Literature	-	M	TOR	[60,93,94,104,127,127]
Hylorina sylvatica	*Hylorina*	Batrachylidae	Photo, literature	FML 16202	LO	ImageJ	FML3667	?	SEMAQ	[34,89,128]
Limnomedusa macroglossa	*Limnomedusa*	Alsodidae	Photo, literature	FML 26369	LO	ImageJ	FML7009	?	TER	[23,37,129,130]
Megaelosia goeldii	*Megaelosia*	Hylodidae	Photo, literature	MZUSP 36455	LO	Literature	-	M	TOR	[3,109,131–133]
Phantasmarana apuana	*Phantasmarana*	Hylodidae	Photo, literature	MTR 10828	LO	Literature	-	M	TOR	[132,134]
Phantasmarana boticariana	*Phantasmarana*	Hylodidae	Photo	CHRP 2826	LO	Literature	-	F	TOR	[132,135]
Phantasmarana jordanensis	*Phantasmarana*	Hylodidae	Literature	-	LO	ImageJ, figure	-	F	TOR	[68,132,136,137]
Phantasmarana massarti	*Phantasmarana*	Hylodidae	Photo, literature	ZUFABC DDBN 40	LO	Literature	-	M	TOR	[132,133]
Phantasmarana tamuia	*Phantasmarana*	Hylodidae	Literature	-	LO	Literature	-	M	TOR	[138]
Thoropa lutzi	*Thoropa*	Cycloramphidae	Literature	-	ST	Literature	-	M	SAX	[70,139–141]
Thoropa megatympanum	*Thoropa*	Cycloramphidae	Photo, literature	DZSJRP 2416.1	ST	ImageJ	ZUEC 15938	M	SAX	[60,70,142,143]
Thoropa miliaris	*Thoropa*	Cycloramphidae	Photo	MZUSP 79966; MZUSP 79993	ST	ImageJ	MNRJ 24979	M	SAX	[52,60,70,139,143,144]
Thoropa petropolitana	*Thoropa*	Cycloramphidae	Photo, literature	MZUSP 79886	ST	ImageJ	MZUSP A-131573	M	SAX	[3,70,73,139,140]
Thoropa saxatilis	*Thoropa*	Cycloramphidae	Literature	-	ST	ImageJ, figure	-	M	SAX	[70,140,145]
Thoropa taophora	*Thoropa*	Cycloramphidae	Literature	-	ST	ImageJ, figure	-	M	SAX	[64,70,139,144,146]

[1] GM: geometric morphometrics; [2] LM: linear measurements.

References

1. Veloso, A.; Díaz, N.; Iturra, P.; Penna, M. Descripción de Una Nueva Especie de Telmatobino Del Género Alsodes (Amphibia, Leptodactylidae) de La Cordillera de Nahuelbuta (Sur de Chile). *Medio Ambiente* **1981**, *5*, 72–77.

2. Díaz, N.F.; Valencia, J. Microhabitat Utilization by Two Leptodactylid Frogs in the Andes of Central Chile. *Oecologia* **1985**, *66*, 353–357.

3. Wassersug, R.J.; Heyer, W.R. A Survey of Internal Oral Features of Leptodactyloid Larvae (Amphibia: Anura). *Smithsonian Contributions to Zoology* **1988**, 1–99, doi:10.5479/si.00810282.457.

4. Lavilla, E.O. Estructura Del Condrocráneo y Esqueleto Visceral de Larvas de Alsodes Barrioi (Anura: Leptodactylidae). *Acta Zoologica Lilloana* **1992**, *XLII*, 13–17.

5. Barrasso, D.A.; Alcade, L.; Blotto, B.L.; Basso, N.G. Description of the Tadpole of Alsodes Neuquensis Cei, 1976 Published by the British Herpetological Society and Comparison with the Sibling Species A. Gargola Gallardo, 1970 (Amphibia, Anura, Alsodidae). *Herpetological Journal* **2016**, *26*, 21–31.

6. Charrier, A. *Guía de Campo: Anfibios de Los Bosques de La Zona Centro Sur y Patagonia de Chile*; Ed. Corporación Chilena de la Madera: Chile, 2019; ISBN 978-956-8398-12-5.

7. Flores, E.F. Ampliación Del Rango de Distribución y Nuevos Antecedentes Del Hábitat de Dos Especies de Ranas Del Género Alsodes Bell, 1843 (Amphibia, Anura, Alsodidae) En La Cordillera de Nahuelbuta, Sur de Chile. *Biodiversity and Natural History* **2018**, *4*, 1–7.

8. Formas, J.R.; Núñez, J.; Cuevas, C. Identidad de La Rana Austral Chilena Eupsophus Coppingeri (Amphibia, Anura, Neobatrachia): Evidencias Morfológicas, Cromosómicas y Moleculares. *Revista Chilena de Historia Natural* **2008**, *81*, 3–20, doi:10.4067/s0716-078x2008000100001.

9. Pillado, M.S.; Alonso, C.A.; Úbeda, C.A. La Larva de Alsodes Gargola Gallardo, 1970 (Leptodactylidae, Telmatobiinae). *Alytes* **2000**, *18*, 62–72.

10. Mella-Romero, J.; Jorge, M.; Muñoz, C.; Correa, C. Range Extension of Catedral Spiny-Chest Frog, Alsodes Gargola Gallardo, 1970: A Record in an Unknown Road of the Chilean Patagonia. *Gayana* **2022**, *86*, 22–29.

11. Cuevas, C.C.; Formas, J.R. A New Species of Alsodes (Amphibia, Anura, Leptodactylidae) from Central Chile. *Amphibia-Reptilia* **2001**, *22*, 187–198.

12. Charrier, A.; Mora, M.; Correa, C.; Palma, E. *Monitoreo y Conservación de Anfibios Alto Andinos de La Región Metropolitana*; América LTDA, 2017;

13. Cuevas, C.C.; Formas, J.R. A New Frog of the Genus Alsodes (Leptodactylidae) from the Tolhuaca National Park, Andes Range, Southern Chile. *Amphibia Reptilia* **2005**, *26*, 39–48, doi:10.1163/1568538053693288.

14. Formas, J.R. Renacuajos Del Grupo Monticola. *Boletin de la Sociedad de Biologia de Concepcion* **1975**, *XLIX*, 231–237.

15. Busse, K. Zur Morphologie Und Biologie von Telmatobius Montanus LATASTE 1902, Nebst Beschreibung Seiner Larve (Amphibia: Leptodactylidae). *Amphibia-Reptilia* **1980**, *1*, 113–125.

16. Araya, S.; Riveros, Y.E. Ampliación Del Rango de Distribución Geográfica de Alsodes Montanus (Amphibia, Leptodactylidae) a La Región de O'Higgins. *Boletín Museo Nacional de Historia Natural* **2008**, *57*, 117–123, doi:10.54830/bmnhn.v57.2008.250.

17. Cei, J.M. Remarks on Some Neotropical Amphibians of the Genus Alsodes from Southern Argentina. *Atti della Societa Italiana di Scienze Naturali e del Museo Civico di Storia Naturale di Milano* **1976**, *117*, 159–164.

18. Cei, J.M. *Batracios de Chile*; Universidad de Chile, 1962; Vol. Vol. 1;

19. Cuevas, C. Análisis Taxonómico de Alsodes Nodosus (Duméril & Bibron, 1841) (Amphibia, Neobatrachia): Antecedentes Morfológicos y Moleculares. **2013**, 1–138.

20. Formas, J.R.; Cuevas, C.C. The Tadpole of Alsodes Cf. Norae (Anura: Alsodidae) with Comments on the Diagnosis of the Genus Alsodes. *Salamandra* **2017**, *53*, 131–136.

21. Cuevas, C. A New Species of the Genus Alsodes (Anura: Neobatrachia) from the Nothofagus Forest, Coastal Range, Southern Chile, Identified by Its Karyotype. *Zootaxa* **2008**, *1771*, 43–53, doi:10.5281/zenodo.182136.

22. Cei, A.J.M.; Roig, V.G. The Systematic Status and Biology of Telmatobius Montanus Lataste (Amphibia , Leptodactylidae). *Copeia* **1965**, *1965*, 421–425.

23. Kehr, A.; Williams, J. *Larvas de Anuros de La República Argentina*; Especial), C. de H. (Serie, Ed.; Asociacion Herpetologica Argentina, 1990;

24. Correa, C.; Pastenes, L.; Iturra, P.; Calderón, P.; Vásquez, D.; Lam, N.; Salinas, H.; Méndez, M.A. Confirmation of the Presence of Alsodes Pehuenche Cei, 1976 (Anura, Alsodidae) in Chile: Morphological, Chromosomal and Molecular Evidence. *Gayana (Concepción)* **2013**, *77*, 125–131, doi:10.4067/s0717-65382013000200006.

25. Álvarez, D.R. Nueva Localidad Para Alsodes Tumultuosus Veloso, Iturra & Galleguillos, 1979 (Amphibia, Alsodidae) En La Cordillera de Los Andes, Region de O'Higgins, Chile. *Biodiversity and Natural History* **2015**, *1*, 1–5.

26. Formas, J.R.; Cuevas, C.; Brieva, L.M. A New Species of Alsodes (Anura: Leptodactylidae) from Cerro Mirador, Cordillera Pelada, Southern Chile. *Proceedings of the Biological Society of Washington* **2002**, *115*, 708–719.

27. Formas, J.R.; Brieva, L. The Tadpoles of Alsodes Vanzolinii and A. Verrucosus (Anura: Leptodactylidae) with Descriptions of Their Internal Oral and Chondrocranial Morphology. *Amphibia Reptilia* **2004**, *25*, 151–164, doi:10.1163/1568538041231238.

28. Formas, J.R. The Identity of the Frog Eupsophus Vanzolinii from Ramadillas, Nahuebuta Range, Southern Chile. *Proceedings of the Biological Society of Washington* **1980**, *93*, 920–927.

29. Diaz, N.F.; Nuñez, H. Nuevo Hallazgo de Alsodes Verrucosus (Philippi, 1902) En Chile y Descripción de Su Larva (Anura: Leptodactylidae). *Bol. Mus. Nac. Hist. Nat. Chile* **1988**, *41*, 87–94.

30. Basso, N.G.; Úbeda, C.A. The Tadpole of Atelognathus Nitoi (Leptodactylidae, Telmatobiinae). *Alytes* **1997**, *15*, 121–126.

31. Echeverría, D.D.; Úbeda, C.A.; Basso, N.G. Microscopía Electrónica de Barrido Del Aparato Bucal y Cavidad Bucofaríngea de La Larva de Atelognathus Nitoi (Anura, Leptodactylidae). *Alytes* **2001**, *19*, 173–182.

32. Úbeda, C.A.; Basso, N.G. The Tadpole of Atelognathus Salai Cei, 1984 (Leptodactylidae: Telmatobiinae). *Amphibia-Reptilia* **2003**, *24*, 112–118.

33. Meriggio, V.; Veloso, A.; Young, S.; Nuñez, H. Atelognathus Jeinimenensis n. Sp. de Leptodactylidae Para El Sur de Chile. *Boletín del Museo Nacional de Historia Natural* **2004**, *53*, 99–123.

34. Grosso, J.; Barrasso, D.A.; Baldo, D.; Nuñez, J.J.; Candioti, F.V. Developmental Diversity in an Endemic Family of Patagonian Frogs (Anura: Batrachylidae). *Biological Journal of the Linnean Society* **2022**, *136*, 240–263, doi:10.1093/biolinnean/blac032.

35. Úbeda, C.; Zagarese, H.; Diaz, M.; Pedrozo, F. First Steps towards the Conservation of the Microendemic Patagonian Frog Atelognathus Nitoi. *Oryx* **1999**, *33*, 59–66, doi:10.1046/j.1365-3008.1999.00035.x.

36. Cei, J.M. The Tadpole of Batrachophrynus Patagonicus Gallardo. *Herpetologica* **1965**, *20*, 242–245.

37. Cei, J.-M.A.M. *Amphibians of Argentina*; Università degli Studi di Firenze: Firenze, 1980; Vol. N. S. Mono;

38. Cuello, M.E.; Úbeda, C.A.; Bello, M.T. Relationship between Morphotypes of Atelognathus Patagonicus (Anura, Neobatrachia) and Environmental Conditions: Evidence and Possible Explanation. *Phyllomedusa* **2008**, *7*, 35–44, doi:10.11606/issn.2316-9079.v7i1p35-44.

39. Cei, J.-M.A.M. The Patagonian Telmatobiid Fauna of the Volcanic Somuncura Plateau of Argentina. *Journal of Herpetology* **1969**, *3*, 1–18.

40. Formas, J.R. Descriptions of Batrachyla (Amphibia, Anura, Leptodactylidae) Tadpoles. *Journal of Herpetology* **1976**, *10*, 221, doi:10.2307/1562983.

41. Atalah, A.; Sielfeld, W.K. Presencia de Batrachyla Antartandica Barrio En Magallanes. *Anales del Instituto de la Patagonia* **1976**, *7*, 168–170.

42. Cuevas, C.C.; Cifuentes, S.L. Amphibia, Anura, Ceratophryidae, Batrachyla Leptopus Bell, 1843: New Records Updating and Geographic Distribution Map, Chile. *Check List* **2010**, *6*, 633–636, doi:10.15560/6.4.633.

43. Cei, J.M.; Capurro, L. Biología y Desarrollo de Eupsophus Taeniatus Girard. *Investigaciones Zoológicas Chilenas* **1958**, *IV*, 159–182.

44. Basso, N.G.; Úbeda, C.A.; Bunge, M.M.; Martinazzo, L.B. A New Genus of Neobatrachian Frog from Southern Patagonian Forests, Argentina and Chile. *Zootaxa* **2011**, 31–44, doi:10.11646/zootaxa.3002.1.3.

45. Toscano, N.P.; Alecrim De Freitas, N.H.; De Rezende, M.R.C.; Provete, D.B.; Lyra, M.; Haddad, C.F.B.; Rossa-Feres, D.D.C.; Da Silva, F.R. External Morphology and Internal Oral Features of the Tadpole of Crossodactylus Caramaschii (Anura: Hylodidae). *Journal of Herpetology* **2019**, *53*, 263–271, doi:10.1670/18-155.

46. Lacerda, J.V.A.; Montesinos, R.; Zocca, C.; Guimarães, C.S.; Santana, D.J.; Ferreira, R.B. On the Stream-Dwelling Crossodactylus Timbuhy (Anura, Hylodidae): Taxonomy, Natural History, and Geographic Distribution. *Zootaxa* **2022**, *5155*, 564–580, doi:10.11646/zootaxa.5155.4.6.

47. Bastos, R.P.; Pombal, J.P.; Bastos, R.P.; Pombal, J.P. New Species of Crossodactylus (Anura: Leptodactylidae) from the Atlantic Rain Forest of Southeastern Brazil. *Copeia* **1995**, *1995*, 436, doi:10.2307/1446907.

48. Nascimento, L.B.; Cruz, C.A.G.; Feio, R.N. A New Species of Diurnal Frog in the Genus Crossodactylus Duméril and Bibron, 1841 (Anura, Leptodactylidae) from Southeastern Brazil. *Amphibia Reptilia* **2005**, *26*, 497–505, doi:10.1163/156853805774806214.

49. Francioni, E.; Carcerelli, L.C. Descrição Do Girino de Crossodactylus Gaudichaudii Duméril & Bibron, 1841 (Anura, Leptodactylidae). *Mem. Inst. Butantan* **1993**, *55*, 63–67.

50. Weber, L.N.; Caramaschi, U. Descrição Da Morfologia Oral Interna de Larvas Do Gênero Crossodactylus Duméril & Bibron, 1841 (Amphibia, Anura, Leptodactylidae). *Arquivos do Museu Nacional, Rio de Janeiro* **2006**, *64*, 141–149.

51. Silva-Soares, T.; Nogueira-Costa, P.; Júnior, V.N.T.B.; Weber, L.N.; Rocha, C.F.D. The Larva of Crossodactylus Aeneus Müller, 1924: Morphology and Ecological Aspects. *Herpetologica* **2015**, *71*, 46–57, doi:10.1655/HERPETOLOGICA-D-13-00048.

52. Fatorelli, P.; Nogueira-Costa, P.; Rocha, C.F.D. Characterization of Tadpoles of the Southward Portion (Oceanic Face) of Ilha Grande, Rio de Janeiro, Brazil, with a Proposal for Identification Key. *North-Western Journal of Zoology* **2018**, *14*, 171–184.

53. Almeida-Gomes, M.; Hatano, F.H.; Sluys, M.V.; Rocha, C.F.D. Diet and Microhabitat Use by Two Hylodinae Species (Anura, Cycloramphidae) Living in Sympatry and Syntopy in a Brazilian Atlantic Rainforest Area. *Iheringia. Série Zoologia* **2007**, *97*, 27–30, doi:https://doi.org/10.1590/S0073-47212007000100005.

54. Faivovich, J. Comments on the Larvae of the Argentine Species of the Genus. *Alytes* **1998**, *16*, 61–67.

55. Bastiani, M.V.I. de; Garcia, P.C. de A.; Lucas, E.M. Crossodactylus Schmidti Gallardo, 1961 (Anura: Hylodidae) in Santa Catarina State, Southern Brazil: A New Record and Comments on Its Conservation Status. *Check List* **2012**, *8*, 262–263.

56. Caldart, V.M.; Iop, S.; Sá, F.P. de; Rocha, M.C. da; Arruda, J.L.S. de; Santos, T.G. dos; Cechin, S.Z. New Records of Crossodactylus Schmidti Gallardo, 1961 (Anura: Hylodidae) for the State of Rio Grande Do Sul, Brazil, with Data on Morphometry and an Updated Geographic Distribution Map. *Check List* **2013**, *9*, 1552–1555.

57. Pimenta, B.V.S.; Cruz, C.A.G.; Caramaschi, U. Taxonomic Review of the Species Complex of Crossodactylus Dispar A. Lutz, 1925 (Anura, Hylodidae). *Arquivos de Zoologia* **2014**, *45*, 1–33, doi:https://doi.org/10.11606/issn.2176-7793.v45i1p1-33.

58. Caramaschi, U.; Kisteumarcher, G. O Girino de Crossodactylus Trachystomus (Reinhardt e Luetken, 1862) (Anura, Leptodactylidae). *Rev. Brasil. Biol.* **1989**, *49*, 237–239.

59. Caramaschi, U.; Sazima, I. Uma Nova Espécie de Crossodactylus Da Serra Do Cipó, Minas Gerais, Brasil (Amphibia, Leptodactylidae). *Revista Brasileira de Zoologia* **1985**, *3*, 43–49, doi:10.1590/s0101-81751985000100005.

60. Pezzuti, T.L.; Leite, F.S.F.; Rossa-Feres, D.D.C.; Garcia, P.C.A. The Tadpoles of the Iron Quadrangle, Southeastern Brazil: A Baseline for Larval Knowledge and Anuran Conservation in a Diverse and Threatened Region. *South American Journal of Herpetology* **2021**, *22*, 1–109, doi:10.2994/SAJH-D-20-00042.1.

61. Pimenta, B.V.S.; Wachlevski, M.; Cruz, C.A.G. Morphological and Acoustical Variation, Geographic Distribution, and Conservation Status of the Spinythumb Frog Crossodactylus Bokermanni Caramaschi and Sazima, 1985 (Anura, Hylodidae). *Journal of Herpetology* **2008**, *42*, 481–492, doi:10.1670/07-164.1.

62. Pimenta, B.V.S.; Caramaschi, U.; Cruz, C.A.G. Synonymy of Crossodactylus Bokermanni Caramaschi & Sazima, 1985 with Crossodactylus Trachystomus (Reinhardt & Lütken, 1862) and Description of a New Species from Minas Gerais, Brazil (Anura: Hylodidae). *Zootaxa* **2015**, *3955*, 65–82, doi:10.11646/zootaxa.3955.1.3.

63. Vidigal, I.; De Carvalho, T.R.; Clemente-Carvalho, R.B.G.; Giaretta, A.A. Vocalizations, Tadpole, and Natural History of Crossodactylus Werneri Pimenta, Cruz & Caramaschi, 2014 (Anura: Hylodidae), with Comments on Distribution and Intraspecific Variation. *Zootaxa* **2018**, *4388*, 61–75, doi:10.11646/zootaxa.4388.1.4.

64. de Sá, F.P.; Haddad, C.F.B.; Gray, M.M.; Verdade, V.K.; Thomé, M.T.C.; Rodrigues, M.T.; Zamudio, K.R. Male-male Competition and Repeated Evolution of Terrestrial Breeding in Atlantic Coastal Forest Frogs*. *Evolution* **2020**, *74*, 459–475, doi:10.1111/evo.13879.

65. Verdade, V.K.; Rodrigues, M.T. A New Species of Cycloramphus (Anura, Leptodactylidae) from the Atlantic Forest, Brazil. *Herpetologica* **2003**, *59*, 513–518, doi:https://doi.org/10.1655/02-78.

66. Verdade, V.K. Relações Filogenéticas Entre as Espécies Dos Gêneros Cycloramphus Tschudi 1838 e Zachaenus Cope 1866 (Anura, Leptodactylidae). **2005**, 241–241.

67. Verdade, V.K.; Almeida-Silva, D.; Cassimiro, J.; Rodrigues, M.T. Rediscovering Cycloramphus Bandeirensis (Anura: Cycloramphidae): Natural History and Breeding Biology of a Vulnerable Species with a Variant Reproductive Mode. *Phyllomedusa: Journal of Herpetology* **2019**, *18*, 159–175, doi:10.11606/issn.2316-9079.v18i2p159-175.

68. Heyer, W.R. Variation and Systematics of Frogs of the Genus Cycloramphus (Amphibia: Leptodactylidae). *Arquivos de Zoologia* **1983**, *30*, 235–339.

69. Heyer, W.R.; Rand, A.S.; Cruz, C.A.G.; Peixoto, O.L.; Nelson, C.E. Frog of Boracéia. *Arquivos de Zoologia* **1990**, *31*, 1–410.

70. Dias, P.H. dos S.; Vera Candioti, F.; Sabbag, A.F.; Colaço, G.; da Silva, H.R.; Haddad, C.F.B.; de Carvalho-e-Silva, A.M.P.T.; Grant, T. Life on the Edge: Tadpoles of Cycloramphidae (Amphibia; Anura), Anatomy, Systematics, Functional Morphology, and Comments on the Evolution of Semiterrestrial Tadpoles. *Journal of Zoological Systematics and Evolutionary Research* **2021**, *59*, 1297–1321, doi:10.1111/jzs.12483.

71. Colaço, G.; Batista, M.; Limp, G.; da Silva, H.R. The Tadpole of an Insular Population of Cycloramphus Boraceiensis Heyer, 1983 (Anura: Cycloramphidae) with a Review of Larval Descriptions for Species in the Genus. *Papeis Avulsos de Zoologia* **2021**, *61*, doi:10.11606/1807-0205/2021.61.48.

72. Verdade, V.K.; Almeida-Silva, D.; Rodrigues, M.T. The Endotrophic Nidicolous Tadpole of Cycloramphus Eleutherodactylus (Miranda-Ribeiro) (Anura: Cycloramphidae). *Zootaxa* **2023**, *5254*, 287–294, doi:10.11646/zootaxa.5254.2.8.

73. Wassersug, R.J.; Heyer, W.R. Morphological Correlates of Subaerial Existence in Leptodactylid Tadpoles Associated with Flowing Water. *Canadian Journal of Zoology* **1983**, *61*, 761–769, doi:10.1139/z83-101.

74. Da Silva, H.R.; Ouvernay, D. A New Species of Stream-Dwelling Frog of the Genus Cycloramphus (Anura, Cycloramphidae) from the State of Rio de Janeiro, Brazil. *Zootaxa* **2012**, 49–60, doi:10.11646/zootaxa.3407.1.4.

75. Lima, A.M.X.; Garey, M.V.; Noleto, R.B.; Verdade, V.K. Natural History of the Lutz's Frog Cycloramphus Lutzorum Heyer, 1983 (Anura: Cycloramphidae) in the Brazilian Atlantic Forest: Description of the Advertisement Call, Tadpole, and Karyotype. *Journal of Herpetology* **2010**, *44*, 360–371, doi:10.1670/08-185.1.

76. Nunes-De-Almeida, C.H.L.; Zamudio, K.R.; Toledo, L.F. The Semiterrestrial Tadpole of Cycloramphus Rhyakonastes Heyer, 1983 (Anura, Cycloramphidae). *Journal of Herpetology* **2016**, *50*, 289–294, doi:10.1670/14-117.

77. Heyer, W.R.; Crombie, R.I. Natural History Notes on Craspedoglossa Stejnegeri and Thoropa Petropolitana (Amphibia: Salientia, Leptodactylidae). *Journal of the Washington Academy of Sciences* **1979**, *69*, 17–20.

78. Lavilla, E.O. Condrocráneo y Esqueleto Visceral En Larvas de Cycloramphus Stejnegeri (Leptodactylidae). *Amphibia-Reptilia* **1991**, *12*, 33–38.

79. Heyer, W.R. Notes on the Frog Genus Cycloramphus (Amphibia: Leptodactylidae), with Descriptions of Two New Species. *Proceedings of the Biological Society of Washington* **1983**, *96*, 548–559.

80. Nuñez, J.J.; Rabanal, F.E.; Formas, J.R. Description of a New Species of Eupsophus (Amphibia: Neobatrachia) from the Valdivian Coastal Range, Southern Chile: An Integrative Taxonomic Approach. *Zootaxa* **2012**, 53–68, doi:10.11646/zootaxa.3305.1.3.

81. Vera Candioti, M.F.; Úbeda, C.; Lavilla, E.O. Morphology and Metamorphosis of Eupsophus Calcaratus Tadpoles (Anura: Leptodactylidae). *Journal of Morphology* **2005**, *264*, 161–177, doi:10.1002/jmor.10320.

82. Nuñez, J.J.; Úbeda, C.A. The Tadpole of Eupsophus Nahuelbutensis (Anura: Neobatrachia): External Morphology, Chondrocranium, and Comments on Its Natural History. *Zootaxa* **2009**, *2126*, 58–68.

83. Formas, J.R.; Vera, M.I. The Status of Two Chilean Frogs of the Genus Eupsophus (Anura: Leptodactylidae). *Proceedings of the Biological Society of Washington* **1982**, *95*, 594–601.

84. Formas, J.R. The Tadpole of Eupsophus Calcaratus in Southern Chile. *Journal of Herpetology* **2009**, *23*, 195–197.

85. Formas, J.R. A New Species of Eupsophus (Amphibia: Anura: Leptodactylidae) from Southern Chile. *Proceedings of the Biological Society of Washington* **1989**, *102*, 568–576.

86. Vera Candioti, M.F.; Nuñez, J.J.; Úbeda, C. Development of the Nidicolous Tadpoles of Eupsophus Emiliopugini (Anura: Cycloramphidae) until Metamorphosis, with Comments on Systematic Relationships of the Species and Its Endotrophic Developmental Mode. *Acta Zoologica* **2011**, *92*, 27–45, doi:10.1111/j.1463-6395.2010.00448.x.

87. Ortiz, J.C.; Vidal, H.I. Una Nueva Especie de Leptodactylidae (Eupsophus) de La Cordillera de Nahuelbuta (Chile). *Acta Zoológica Lilloana* **1991**, *41*, 75–79.

88. Formas, J.R.; Pugín, E. Tadpoles of Hylorina Sylvatica, Eupsophus Vittatus, and Bufo Rubropunctatus in Southern Chile. *Herpetologica* **1978**, *34*, 355–358.

89. Cárdenas-Rojas, D.R.; Veloso, A.; De Sá, R.O. The Tadpole of Eupsophus Queulensis (Anura, Cycloramphidae). *Alytes* **2007**, *25*, 45–54.

90. Ibarra-Vidal, H.; Ortiz, J.C.; Torres-Pérez, F. Eupsophus Septentrionalis n. Sp. a New Leptodactulid Amphibian from Central Chile. *Boletin de la Sociedad de Biologia de Concepcion* **2004**, *75*, 91–102.

91. Formas, R. The Tadpole of Eupsophus Vertebralis (Anura: Leptodactylidae). *Herpetologica* **1992**, *48*, 115–119.

92. Pombal, J.P.; Feio, R.N.; Haddad, C.F.B. A New Species of Torrent Frog Genus Hylodes (Anura: Leptodactylidae) from Southeastern Brazil. *Herpetologica* **2002**, *58*, 462–471, doi:10.1655/0018-0831(2002)058[0462:ANSOTF]2.0.CO;2.

93. Montesinos, R.; Carvalho, A.L.G.; Silva, H.R.D.; Anganoycriollo, M.; Dias, P.H.D.S. The Tadpole of Hylodes Perere Silva & Benmaman 2008 (Anura: Hylodidae). *Zootaxa* **2022**, *5219*, 388–396, doi:10.11646/zootaxa.5219.4.6.

94. Canedo, C. Revisão Taxonômica de Hylodes Fitzinger, 1826 (Anura, Hylodidae), Universidade Federal do Rio de Janeiro, 2008.

95. Bokermann, W.C.A. Girinos de Anfíbios Brasileiros - 2 (Amphibia, Salientia). *Rev. Brasil. Biol.* **1963**, *23*, 349–353.

96. Almeida-Silva, D.; Hipolito, M.; Martins, A.M.C.R.P. da F.; Batista, B.L.; Pedron, T.; Dias, G.M.; Verdade, V.K. Frog Hepatic Health and Metal Pollution: An Assemblage-Level Approach. *SSRN - preprint* **2024**, 1–52, doi:http://dx.doi.org/10.2139/ssrn.4580718.

97. Nogueira-Costa, P.; Silva-Soares, T.; Weber, L.N.; Carvalho-E-silva, A.M.P.T.D. Redescription of Tadpole of the Hylodid Frog Hylodes Asper (Müller, 1924). *Zootaxa* **2010**, *68*, 65–68, doi:10.11646/zootaxa.2521.1.5.

98. Pirani, R.M.; Pezzuti, T.L.; Motta, A.P.; Feio, R.N. The Tadpole of Hylodes Babax Heyer, 1982 (Amphibia, Anura, Hylodidae). *Zootaxa* **2011**, *1930*, 64–68, doi:10.11646/zootaxa.2800.1.5.

99. Heyer, W.R. Two New Species of the Frog Genus Hylodes from Caparaó, Minas Gerais, Brasil (Amphibia: Leptodactylidae). *Proceedings of the Biological Society of Washington* **1982**, *95*, 377–385.

100. Malagoli, L.R.; Sá, F.P. de; Canedo, C.; Haddad, C.F.B. The Tadpole of Hylodes Caete (Anura: Hylodidae), an Endemic Atlantic Rainforest Torrent Frog of Brazil. *Zootaxa* 2020, *4852*, 594–599.

101. Malagoli, L.R.; De Sá, F.P.; Canedo, C.; Haddad, C.F.B. A New Species of Hylodes (Anura, Hylodidae) from Serra Do Mar, Southeastern Brazil: The Fourth with Nuptial Thumb Tubercles. *Herpetologica* **2017**, *73*, 136–147, doi:10.1655/HERPETOLOGICA-D-16-00069.

102. Nogueira-Costa, P.; Siqueira, C.C.; Vrcibradic, D.; Weber, L.N.; Rocha, C.F.D. The Tadpole of the Hylodid Frog Hylodes Charadranaetes Heyer and Cocroft, 1986. *Zootaxa* **2010**, 65–68, doi:10.11646/zootaxa.2680.1.6.

103. Pavan, D.; Narvaes, P.; Rodrigues, M.T. A New Species of Leptodactylid Frog from the Atlantic Forests of Southeastern Brazil with Notes on the Status and the Speciation of the Hylodes Species Groups. *Papeis Avulsos de Zoologia* **2001**, *41*, 407–425.

104. Weber, L.N.; Caramaschi, U. A Survey of the Internal Oral Morphology in Larvae of the Genus Hylodes Fitzinger, 1826 (Amphibia, Anura, Hylodidae). *Zootaxa* **2013**, *3635*, 557–568, doi:10.11646/zootaxa.3635.5.5.

105. Canedo, C.; Pombal, J.P. Two New Species of Torrent Frog of the Genus Hylodes (Anura, Hylodidae) with Nuptial Thumb Tubercles. *Herpetologica* **2007**, *63*, 224–235, doi:10.1655/0018-0831(2007)63[224:TNSOTF]2.0.CO;2.

106. Laia, R.C.; Fatorelli, P.; Hatano, F.H.; Duarte Rocha, C.F. Tadpole of Hylodes Fredi (Anura; Hylodidae), a Frog Endemic to an Atlantic Forest Island (Ilha Grande, Rio de Janeiro State), Brazil. *Zootaxa* **2010**, 62–64, doi:10.11646/zootaxa.2640.1.5.

107. Nogueira-Costa, T.R.; Lingnau, R.; Toledo, L.F. The Tadpole of the Brazilian Torrent Frog Hylodes Heyeri (Anura; Hylodidae). *Zootaxa* **2009**, *68*, 66–68, doi:10.11646/zootaxa.2222.1.6.

108. Haddad, C.F.B.; Pombal, J.P.; Bastos, R.P. New Species of Hylodes from the Atlantic Forest of Brazil (Amphibia: Leptodactylidae). *Copeia* **1996**, *1996*, 965–969, doi:10.2307/1447659.

109. Lutz, A. Contribution to the Knowledge of Brazilian Batrachians: Taxonomy and Biology of the Elosiinae. *Memórias do Instituto Oswaldo Cruz* **1930**, *XXIV*, 223–244.

110. de Sá, F.P.; Canedo, C.; Lyra, M.L.; Haddad, C.F.B. A New Species of Hylodes (Anura, Hylodidae) and Its Secretive Underwater Breeding. *Herpetologica* **2015**, *71*, 58–71.

111. Gomes, F.B.R.; Provete, D.B.; Martins, I.A. Description of the Tadpole of Hylodes Magalhaesi (Bokermann, 1964) (Anura: Hylodidae). *Journal of Herpetology* **2012**, *46*, 614–619, doi:10.1670/11-039.

112. Bokermann, W.C.A. Una Nueva Especies de Elosia de La Serra Da Mantiqueira, Brasil. *Neotropica* **1964**, *10*, 102–107.

113. Nogueira-Costa, P.; Wachlevski, M. The Tadpole of Hylodes Meridionalis (Mertens, 1927), a Lotic Stream Anura from the Atlantic Rainforest of Brazil. *Zootaxa* **2015**, *4032*, 199–202, doi:10.11646/zootaxa.4032.2.5.

114. Lingnau, R.; Zank, C.; Colombo, P.; Kwet, A. Vocalization of Hylodes Meridionalis (Mertens 1927) (Anura, Hylodidae) in Rio Grande Do Sul, Brazil, with Comments on Nocturnal Calling in the Family Hylodidae. *Studies on Neotropical Fauna and Environment* **2013**, *48*, 76–80, doi:10.1080/01650521.2013.788285.

115. Wogel, H.; Abrunhosa, P.A.; Weber, L.N. The Tadpole, Vocalizations and Visual Displays of Hylodes Nasus (Anura: Leptodactylidae). *Amphibia Reptilia* **2004**, *25*, 219–227, doi:10.1163/1568538041231184.

116. Machado, A.D.O.; Winck, G.; Dorigo, T.A.; Rocha, C.F.D. Diet, Diel Activity Pattern, Habitat Use, and Reproductive Effort of Hylodes Nasus (Anura: Hylodidae) in One of the World's Largest Urban Parks (Tijuca National Park), Southeastern Brazil. *South American Journal of Herpetology* **2016**, *11*, 127–135, doi:10.2994/SAJH-D-16-00004.1.

117. Bilate, M.; Nogueira-Costa, P.; Weber, L.N. The Tadpole of the Hylodid Frog Hylodes Ornatus (Bokermann, 1967), Including Chondrocranium Description, and Advertisement Call. *Zootaxa* **2012**, *66*, 60–66, doi:10.11646/zootaxa.3249.1.6.

118. Bokermann, W.C.A. Una Nueva Especie de Elosia de Itatiaia, Brasil (Amphibia, Leptodactylidae). *Neotropica* **1967**, *13*, 135–137.

119. Sazima, I.; Bokermann, W. Anfíbios Da Serra Do Cipó, Minas Gerais, Brasil. 5: Hylodes Otavioi Sp. n. (Anura, Leptodactylidae). *Revista Brasileira de Biologia* **1982**, *42*, 767–771.

120. Silva, H.R. da; Benmaman, P. Uma Nova Espécie de Hylodes Fitzinger Da Serra Da Mantiqueira, Minas Gerais, Brasil (Anura: Hylodidae). *Revista Brasileira de Zoologia* **2008**, *25*, 89–99, doi:10.1590/S0101-81752008000100013.

121. Haddad, C.F.B.; Garcia, P.C.A.; Jr, J.P.P. Redescrição de Hylodes Perplicatus (Miranda-Ribeiro, 1926) (Amphibia, Anura, Leptodactylidae). *Arquivos do Museu Nacional, Rio de Janeiro* **2003**, *61*, 245–254.

122. Menegucci, R.C.; Muscat, E.; Tanaka, R.M.; Stuginski, D. Predation on Hylodes Phyllodes (Anura: Hylodidae) by the Harvestman Heteromitobates Discolor (Arachnida: Opiliones). *Phyllomedusa* **2020**, *19*, 121–124, doi:10.11606/issn.2316-9079.v19i1p121-124.

123. Alencar, L.R.V.; Gaiarsa, P.; Gennari, D.; Martins, M. Hylodes Phyllodes (Amphibia, Anura, Hylodidae): Nocturnal Calling Behavior. *Herpetologia Brasileira* **2012**, *1*, 82–83.

124. Nogueira-Costa, P.; Weber, L.N.; Wogel, H.; Salles, R.D.O.L.; Bilate, M. A Description of the Tadpoles of Hylodes Pipilans Canedo and Pombal, 2007: An Endemic Species of the Atlantic Forest of Brazil. *Herpetological Conservation and Biology* **2019**, *14*, 370–379.

125. Carmo, L.F.; Folly, M.; Malagoli, L.R. An Update of the Geographical Distribution of Hylodes Pipilans (Canedo & Pombal, 2007), an Endemic Torrent Frog of the Brazilian Atlantic Forest, with Comments on Its Conservation. *Neotropical Biodiversity* **2022**, *8*, 131–135, doi:10.1080/23766808.2022.2049171.

126. Haddad, C.F.B.; Pombal Jr., J.P. A New Species of Hylodes from Southeastern Brazil (Amphibia: Leptodactylidae). *Herpetologica* **1995**, *51*, 279–286.

127. Nascimento, L.B.; Pombal, J.P.; Haddad, C.F.B. A New Frog of the Genus Hylodes (Amphibia: Leptodactylidae) from Minas Gerais, Brazil. *Journal of Zoology* **2001**, *254*, 421–428, doi:10.1017/S0952836901000917.

128. Úbeda, C.; Moncada, M.; Jara, F. Fenología Del Ciclo de Vida de La Rana Esmeralda Hylorina Sylvatica Bell 1843 (Anura, Batrachylidae) En Un Humedal Semipermanente Del Noroeste de La Patagonia Argentina. *Boletín Chileno de Herpetología* **2022**, *9*, 1–11.

129. Guerra, V.; Lingnau, R.; Barreto, B. de S.; Kwet, A.; Bastos, R.P. Vocalizations of Limnomedusa Macroglossa (Amphibia: Anura: Alsodidae). *Salamandra* **2018**, *54*, 97–100.

130. Alcalde, L.; Blotto, B.L. Chondrocranium, Cranial Muscles and Buccopharyngeal Morphology on Tadpoles of the Controversial Leptodactylid Frog Limnomedusa Macroglossa (Anura: Leptodactylidae). *Amphibia Reptilia* **2006**, *27*, 241–253, doi:10.1163/156853806777239959.

131. da Silva, D. das N.; da Rosa, F.C.B.; de Carvalho-E-Silva, A.M.P.T. Ontogeny and Behavioural Aspects of the Tadpoles of Megaelosia Goeldii (Baumann, 1912) (Amphibia, Anura, Hylodidae). *Herpetology Notes* **2018**, *11*, 629–639.

132. Sichieri, G.; Moroti, M. de T.; Costa, F.R.; Muscat, E.; Nunes, I. Redescription of the Tadpole of Rare Giant Torrent Frog Megaelosia Boticariana Giaretta & Aguiar, 1998 (Anura, Hylodidae) with Comments on Natural History. *Zootaxa* **2020**, *4896*, 140–144, doi:10.11646/zootaxa.4896.1.9.

133. Giaretta, A.A.; Bokermann, W.C.A.; Haddad, C.F.B. A Review of the Genus Megaelosia (Anura: Leptodactylidae) with a Description of a New Species. *Journal of Herpetology* **1993**, *27*, 276, doi:10.2307/1565148.

134. Pombal, J.P.; Prado, G.M.; Canedo, C. A New Species of Giant Torrent Frog, Genus Megaelosia, from the Atlantic Rain Forest of Espírito Santo, Brazil (Amphibia: Leptodactylidae). *Journal of Herpetology* **2003**, *37*, 453–460, doi:10.1670/220-01a.

135. Giaretta, A.A.; Aguiar Jr., O.A. A New Species of Megaelosia from the Mantiqueira Range, Southeastern Brazil. *Journal of Herpetology* **1998**, *32*, 80–83, doi:10.2307/1565483.

136. Nogueira-Costa, P.; Almeida-Santos, P.; Cruz, C.A.G.; Caramaschi, U. The Giant Tadpoles of Megaelosia Jordanensis (Heyer, 1983). *Zootaxa* **2012**, 86–88, doi:10.11646/zootaxa.3581.1.4.

137. Verdade, V.K.; Rodrigues, M.T. On the Identity of Cycloramphus Jordanensis Heyer, 1983 (Anura: Cycloramphidae). *Herpetologica* **2008**, *64*, 452–457, doi:10.1655/08-011R2.1.

138. De Sá, F.P.; Condez, T.H.; Lyra, M.L.; Haddad, C.F.B.; Malagoli, L.R. Unveiling the Diversity of Giant Neotropical Torrent Frogs (Hylodidae): Phylogenetic Relationships, Morphology, and the Description of Two New Species. *Systematics and Biodiversity* **2022**, *20*, 1–31, doi:10.1080/14772000.2022.2039318.

139. Bokermann, W.C.A. Notas Sôbre as Espécies de Thoropa Fitzinger (Amphibia, Leptodactylidae). *Anais da Academia Brasileira de Ciências. Rio de Janeiro* **1965**, *37*, 525–537.

140. Sabbag, A.F.; Lyra, M.L.; Zamudio, K.R.; Haddad, C.F.B.; Feio, R.N.; Leite, F.S.F.; Gasparini, J.L.; Brasileiro, C.A. Molecular Phylogeny of Neotropical Rock Frogs Reveals a Long History of Vicariant Diversification in the Atlantic Forest. *Molecular Phylogenetics and Evolution* **2018**, *122*, 142–156, doi:10.1016/j.ympev.2018.01.017.

141. Assis, C.L.; Lacerda, J.V.A.; Guimares, C.S.; Peixoto, M.A.; Luna, M.C.; Feio, R.N. A New Species of Thoropa Cope, 1865 (Anura, Cycloramphidae) from the Serra Da Mantiqueira, Southeast Brazil. *Zootaxa* **2021**, *4995*, 505–522, doi:10.11646/zootaxa.4995.3.6.

142. Caramaschi, U.; Sazima, I. Uma Nova Espécie de Thoropa Da Serra Do Cipó, Minas Gerais, Brasil (Amphibia, Leptodactylidae). *Revista Brasileira de Zoologia* **1984**, *2*, 139–146.

143. Santos, D.L.; Feio, R.N.; Nomura, F. Morphological Characterization and Taxonomic Key for Tadpoles of Brazilian Cerrado. *Biota Neotropica* **2023**, *23*, doi:10.1590/1676-0611-BN-2023-1486.

144. Feio, R.N.; Napoli, M.F.; Caramaschi, U. Considerações Taxonômicas Sobre Thoropa Miliaris (Spix, 1824), Com Revalidação e Redescrição de Thoropa Taophora (Miranda-Ribeiro, 1923) (Amphibia, Anura, Leptodactylidae). *Arquivos do Museu Nacional: Zoologia* **2006**, *64*, 41–60.

145. Cocroft, R.B.; Heyer, W.R. Notes on the Frog Genus Thoropa (Amphibia: Leptodactylidae) with a Description of a New Species (Thoropa Saxatilis). *Proceedings of the Biological Society of Washington* **1988**, *101*, 209–220.

146. Moura, P.H.A.G.; Alves, S.J.D.R.; Sousa, D.D.G.D.S.E.; Correa, C.N.; Nunes, I. Redescription of the Tadpole of Thoropa Taophora (Miranda-Ribeiro) (Anura: Cycloramphidae). *Zootaxa* **2019**, *4656*, doi:10.11646/zootaxa.4656.2.14.

147. Almeida-Silva, D.; Rocha-Barros, V.C. de O.; Ferreira, R.B.; Verdade, V.K. The Tadpole of Zachaenus Carvalhoi Izecksohn, 1983 (Anura: Cycloramphidae). *Zootaxa* **2019**, *4668*, 145–147, doi:10.11646/zootaxa.4668.1.11.

148. Lutz, B. Biologia e Taxonomia de Zachaenus Parvulus. *Boletim do Museu Nacional de Zoologia* **1944**, *17*, 1–65.
