# Supplementary material for: Shape Evolution in Two Acts: Morphological Diversity of Larval and Adult Neoaustraranan Frogs
Source: Animals (Basel). 2024 May 8;14(10):1406. doi: 10.3390/ani14101406 (PMC11117230; doi:10.3390/ani14101406)
Supplement: Supplementary file 1 [file animals-14-01406-s001.zip › TableS2.pdf]

Table S2. Procrustes ANOVA of larval shape variation to explore the effect of genus, family, ecomorphological guild, and developmental stage as factors.

	df	SS	MS	Rsq	F	Z	p-value
*Tadpole' shape ~ Gosner * family*							
Gosner	2	0.017	0.009	0.008	0.51	-0.49	0.67
Family	3	0.496	0.165	**0.219**	98.14	39.72	**<0.01**
Gosner: Family	6	0.071	0.012	0.031	0.70	-0.54	0.70
Residuals	100	168.458	0.017	0.742			
Total	111	226.889					
*Tadpole' shape ~ Gosner * genus*							
Gosner	2	0.017	0.009	0.008	0.63	-0.23	0.59
Genus	12	102.129	0.085	**0.450**	62.19	46.96	**<0.01**
Gosner: Genus	14	0.094	0.007	0.042	0.49	-17.70	0.96
Residuals	83	113.593	0.014	0.501			
Total	111	226.889					
*Tadpole' shape ~ Gosner * ecomorphological guild*							
Gosner	2	0.017	0.009	0.008	0.50	-0.53	0.69
Guild	3	0.439	0.146	**0.193**	84.20	38.40	**<0.01**
Gosner:Guild	5	0.058	0.012	0.026	0.67	-0.59	0.72
Residuals	101	175.489	0.017	0.773			
Total	111	226.889					
