# Supplementary material for: Shape Evolution in Two Acts: Morphological Diversity of Larval and Adult Neoaustraranan Frogs
Source: Animals (Basel). 2024 May 8;14(10):1406. doi: 10.3390/ani14101406 (PMC11117230; doi:10.3390/ani14101406)
Supplement: Supplementary file 1 [file animals-14-01406-s001.zip › TableS3.pdf]

Table S3. Statistics and sample size from the test for allometry for each family, under the null hypothesis of isometry

	N	**% predicted**	***p-value***
Tadpoles (shape ~ log(TL))			
Alsodidae	30	21.19	<0.01
Batrachylidae	11	12.19	0.27
Cycloramphidae	26	12.32	0.04
Hylodidae	45	6.70	0.03
Adults (shape ~ log(SVL))			
Alsodidae	30	10.25	0.06
Batrachylidae	11	36.19	<0.01
Cycloramphidae	26	5.86	0.34
Hylodidae	45	12.10	0.01

[1] TL: total length; [2] SVL: snout-vent length; *N*: number of individuals by family.
